# Automated graded prognostic assessment for patients with hepatocellular carcinoma using machine learning

**DOI:** 10.1007/s00330-024-10624-8

**Published:** 2024-03-27

**Authors:** Moritz Gross, Stefan P. Haider, Tal Ze’evi, Steffen Huber, Sandeep Arora, Ahmet S. Kucukkaya, Simon Iseke, Bernhard Gebauer, Florian Fleckenstein, Marc Dewey, Ariel Jaffe, Mario Strazzabosco, Julius Chapiro, John A. Onofrey

**Affiliations:** 1 Department of Radiology and Biomedical Imaging, Yale University School of Medicine, New Haven, CT, USA.; 2 Charité Center for Diagnostic and Interventional Radiology, Charité - Universitätsmedizin Berlin, Berlin, Germany.; 3 Department of Otorhinolaryngology, University Hospital of Ludwig Maximilians Universität München, Munich, Germany.; 4 Department of Biomedical Engineering, Yale University, New Haven, CT, USA.; 5 Department of Diagnostic and Interventional Radiology, Pediatric Radiology and Neuroradiology, Rostock University Medical Center, Rostock, Germany.; 6 Department of Internal Medicine, Yale University School of Medicine, New Haven, CT, USA.; 7 Department of Urology, Yale University School of Medicine, New Haven, CT, USA.

**Keywords:** Hepatocellular carcinoma, Risk assessment, Magnetic resonance imaging, Medical image processing, Machine learning

## Abstract

**Background:**

Accurate mortality risk quantification is crucial for the management of hepatocellular carcinoma (HCC); however, most scoring systems are subjective.

**Purpose:**

To develop and independently validate a machine learning mortality risk quantification method for HCC patients using standard-of-care clinical data and liver radiomics on baseline magnetic resonance imaging (MRI).

**Methods:**

This retrospective study included all patients with multiphasic contrast-enhanced MRI at the time of diagnosis treated at our institution. Patients were censored at their last date of follow-up, end-of-observation, or liver transplantation date. The data were randomly sampled into independent cohorts, with 85% for development and 15% for independent validation. An automated liver segmentation framework was adopted for radiomic feature extraction. A random survival forest combined clinical and radiomic variables to predict overall survival (OS), and performance was evaluated using Harrell’s C-index.

**Results:**

A total of 555 treatment-naïve HCC patients (mean age, 63.8 years ± 8.9 [standard deviation]; 118 females) with MRI at the time of diagnosis were included, of which 287 (51.7%) died after a median time of 14.40 (interquartile range, 22.23) months, and had median followed up of 32.47 (interquartile range, 61.5) months. The developed risk prediction framework required 1.11 min on average and yielded C-indices of 0.8503 and 0.8234 in the development and independent validation cohorts, respectively, outperforming conventional clinical staging systems. Predicted risk scores were significantly associated with OS (*p* <*.00001* in both cohorts).

**Conclusions:**

Machine learning reliably, rapidly, and reproducibly predicts mortality risk in patients with hepatocellular carcinoma from data routinely acquired in clinical practice.

**Clinical relevance statement:**

Precision mortality risk prediction using routinely available standard-of-care clinical data and automated MRI radiomic features could enable personalized follow-up strategies, guide management decisions, and improve clinical workflow efficiency in tumor boards.

**Key Points:**

- Machine learning enables hepatocellular carcinoma mortality risk prediction using standard-of-care clinical data and automated radiomic features from multiphasic contrast-enhanced MRI.
- Automated mortality risk prediction achieved state-of-the-art performances for mortality risk quantification and outperformed conventional clinical staging systems.
- Patients were stratified into low, intermediate, and high-risk groups with significantly different survival times, generalizable to an independent evaluation cohort.

## Introduction

Liver cancer is the second most frequent cause of cancer-related death in the world [1] and the fifth leading cause in the United States [2]. In contrast to other cancer types, liver cancer incidence and mortality rates are rising [3, 4]. Hepatocellular carcinoma (HCC) is the most prevalent form of primary liver cancer, accounting for 70–85% of total liver cancers globally [5]. Imaging is a critical tool for diagnosing and staging of HCC. Magnetic resonance imaging (MRI) provides high spatial resolution and allows soft-tissue characterization of the liver. The liver imaging reporting and data system (LI-RADS) [6] has been developed for non-invasive diagnosis based on imaging criteria, and in most cases, HCC can be detected and diagnosed using contrast-enhanced multiphasic imaging without an invasive biopsy [7].

Various scoring and staging systems have been proposed for HCC mortality risk quantification and to stratify patients into risk categories [8–14]. However, these systems use only limited one-dimensional tumor size measurements [9–13], do not make use of quantitative imaging biomarkers, and stratify patients into different risk groups based on strict thresholds. Traditional one-dimensional tumor size measurements have major limitations in reflecting viability, actual tumor size, and growth potential [15] and are subject to rater variability. Quantitative image biomarkers, such as radiomic features, can be extracted from regions and volumes of interest from medical imaging data; however, automated segmentation methods are required for integration into clinical practice workloads. Fully automated whole liver segmentation based on deep learning has demonstrated robust, reproducible, and generalizable segmentation performance across disease stages with substantially altered liver morphology and only required processing times in the order of seconds [16].

The aim of this study was twofold: develop and validate an automated method for mortality risk quantification in HCC patients using only routinely available standard-of-care clinical variables and radiomic features derived from automated liver segmentations on baseline multiphasic contrast-enhanced MRI by means of machine learning and use this risk score to stratify patients into low, intermediate, and high-risk groups.

## Materials and methods

### Compliance with ethical standards

This HIPAA-compliant study was approved by the Yale School of Medicine institutional review board with full waiver of consent and conducted in accordance with the declaration of Helsinki.

### Code availability

All code for the methodological implementation and the trained framework is publicly available on GitHub: https://github.com/OnofreyLab/hcc-mortality-risk

### Data availability

Patient data and imaging data used in this paper cannot be shared publicly due to legal reasons.

### Patient inclusion and exclusion

This retrospective study identified all patients with treatment-naïve HCC treated at our institution between the years 2008 and 2019. HCC was either proven by imaging criteria or biopsy confirmation. We included

- all patients >18 years old
- that had multiphasic contrast-enhanced MRI at the time of diagnosis.

We excluded

- all patients with missing clinical information,
- with no triphasic MRI acquisition or
- non-diagnostic MRI.

The patients were randomly sampled into independent cohorts, with 85% for development and 15% for independent validation.

### Clinical data

Clinical data were collected from the hospital’s electronic health record system, and conventional clinical staging scores [8–14] were calculated. Patients were evaluated for extrahepatic metastases on chest computed tomography (CT) and bone scans at the date of diagnosis, and the following laboratory values were collected closest to the date of imaging: alpha-fetoprotein (AFP), total bilirubin, direct bilirubin, serum albumin, international normalized ratio (INR), partial thromboplastin time (PTT), sodium, and creatinine.

### MRI acquisition and radiomics extraction

Multiphasic contrast-enhanced MRI was acquired using a standard institutional imaging protocol involving T1-weighted breath-hold sequences before contrast administration and 12–18 s, 60–70 s, and 3–5 min post-contrast injection for pre-contrast-, late arterial, portal venous-, and delayed-phase images, respectively. The scans were de-identified and downloaded from the picture archiving and communication system (PACS) server. Subsequently, automated image co-registration and whole liver segmentation were performed using a convolutional neural network [17], followed by radiomic feature extraction. Full details on the image processing pipeline can be found in Supplement 1.

### Survival model and graded prognostic assessment

A random survival forest (RSF) [18] is a non-parametric method that uses an ensemble of survival trees to analyze right-censored time-to-event data. In this study, we fit an RSF with overall survival (OS) as the dependent variable and the combination of all clinical- and radiomic variables as independent variables (termed “candidate variables”). OS was defined as the period between the imaging date and the date of death of any cause. Patients were censored either at their last date of follow-up, end-of-observation date, or at the date of liver transplantation. The following RSF settings were used: 1000 estimator trees were grown with a minimum of 10 samples to split an internal node, and 15 samples were required at a leaf node with $\sqrt{n}$ variables to consider when looking for the best split. First, all candidate variables with a Pearson correlation coefficient > 0.9 in the development cohort were excluded to reduce multicollinearity. Second, we fit the RSF on the remaining candidate variables and obtained a ranked list of variable importance scores by ten permutations of random shuffling. Third, we re-fit the final survival model on the 30 topmost important variables. To gain further insight and interpretability of the proposed method, we obtained variable importance scores by ten permutations of random shuffling for all included variables. Our method was implemented in Python (v3.8.11) using the scikit-survival package (v0.16.0). Three risk groups were defined from the risk score predictions of the proposed model using the “rhier” function of the R package “rolr” (v1.0), which uses a hierarchical method by applying ordered logrank tests [19], to stratify patients into low-, intermediate-, and high-risk groups. The risk score cutoffs for each risk group were derived from the development cohort, with a minimum of 25 subjects in each risk group. The same cutoff values were then applied for risk group stratification in the independent validation cohort. For performance evaluation of the proposed model and conventional clinical staging scores, we calculated Harrell’s C-index [20] and the area under the time-dependent receiver operating characteristic curve (AUC) [21] at 1–5 years after the date of imaging.

### Statistical analysis

Statistical analyses were conducted in SPSS (v27), R (v4.1.1), and Python (v3.8.11). *p* values < .05 were considered statistically significant, and Bonferroni correction was used when comparing multiple groups. Cox proportional hazards regression analysis was used to determine the association of the developed risk score and the proposed risk groups with OS. Median OS (mOS) was calculated, and Kaplan-Meier survival curves were plotted for each risk group and compared using a logrank test. The survival rates of the proposed risk groups were calculated at 1, 3, and 5 years after the date of imaging. To assess the generalizability of the risk groups’ survival times to new cohorts, the survival times of each risk group were compared between the development- and validation cohort using a logrank test.

## Results

### Patient characteristics

**Fig. 1:**
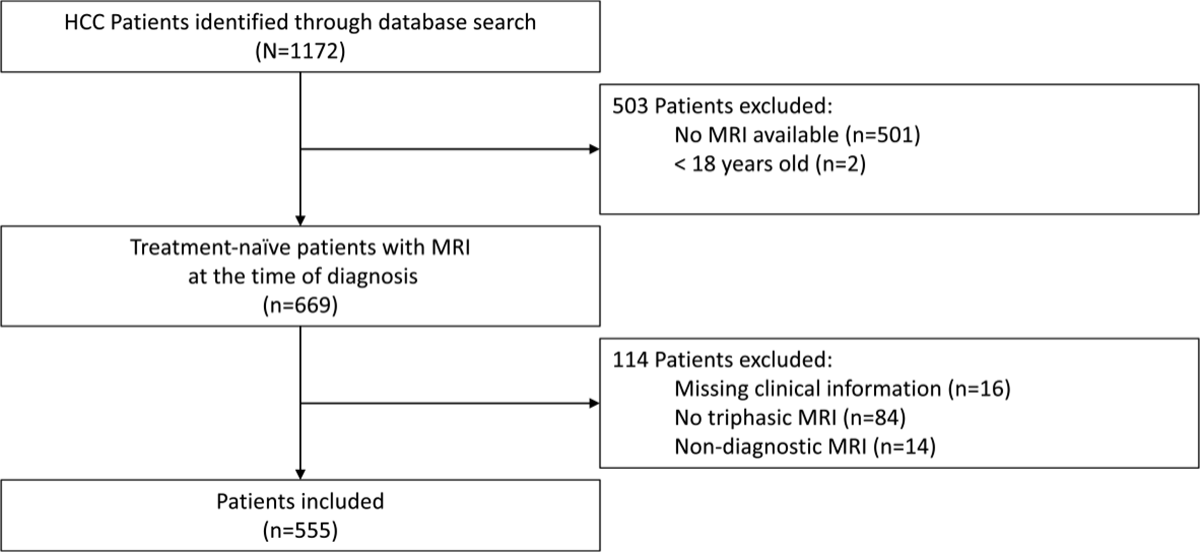
Flowchart of patient inclusion and exclusion. From an institutional database with 1172 patients, 555 patients (118 females, 437 males, 63.8 ± 8.9 years) with imaging- or histopathologically proven treatment-naïve hepatocellular carcinoma and baseline multiphasic contrast-enhanced magnetic resonance imaging at the time of diagnosis were included in the study

A total of 555 patients (mean age, 63.8 years ± 8.9 [standard deviation]; 118 females) with treatment-naïve HCC and multiphasic contrast-enhanced MRI at the time of diagnosis were included in the study. Patients without MRI at baseline (*n* = 501), < 18 years (*n* = 2), missing clinical information (*n* = 16), no triphasic image acquisition (*n* = 84), and non-diagnostic MRI (*n* = 14) were excluded from the study (Fig. 1). Patient baseline characteristics are summarized in Table 1, and MRI parameters are reported in Supplemental Table 1. HCC was either proven by imaging criteria or histopathology.

A total of 287 (51.7%) patients died after a median time of 14.40 months (range, 0.20–97.12 months; interquartile range (IQR), 22.23) after the date of imaging, and patients were followed up for a median of 32.47 months (range: 0.20–118.90 months; IQR: 61.5) after the date of imaging. The median time between the laboratory results and the imaging date was 10 days (IQR, 28.3). First treatments based on the institution’s multidisciplinary tumor board decisions were as follows: 192 (34.6%) patients underwent transarterial chemoembolization, 138 (24.9%) thermal ablation, 82 (14.8%) hepatectomy, 68 (12.3%) a combination of transarterial chemoembolization and thermal ablation, 24 (4.3%) Sorafenib, 24 (4.3%) best supportive care, 20 (3.6%) transarterial radioembolization with Yttrium-90, and lastly 7 (1.3%) liver transplantation. For model development and validation, a total of 471 (85%) patients were randomly allocated to the development cohort and 84 (15%) to the independent validation cohort.

### Survival model

**Fig. 2:**
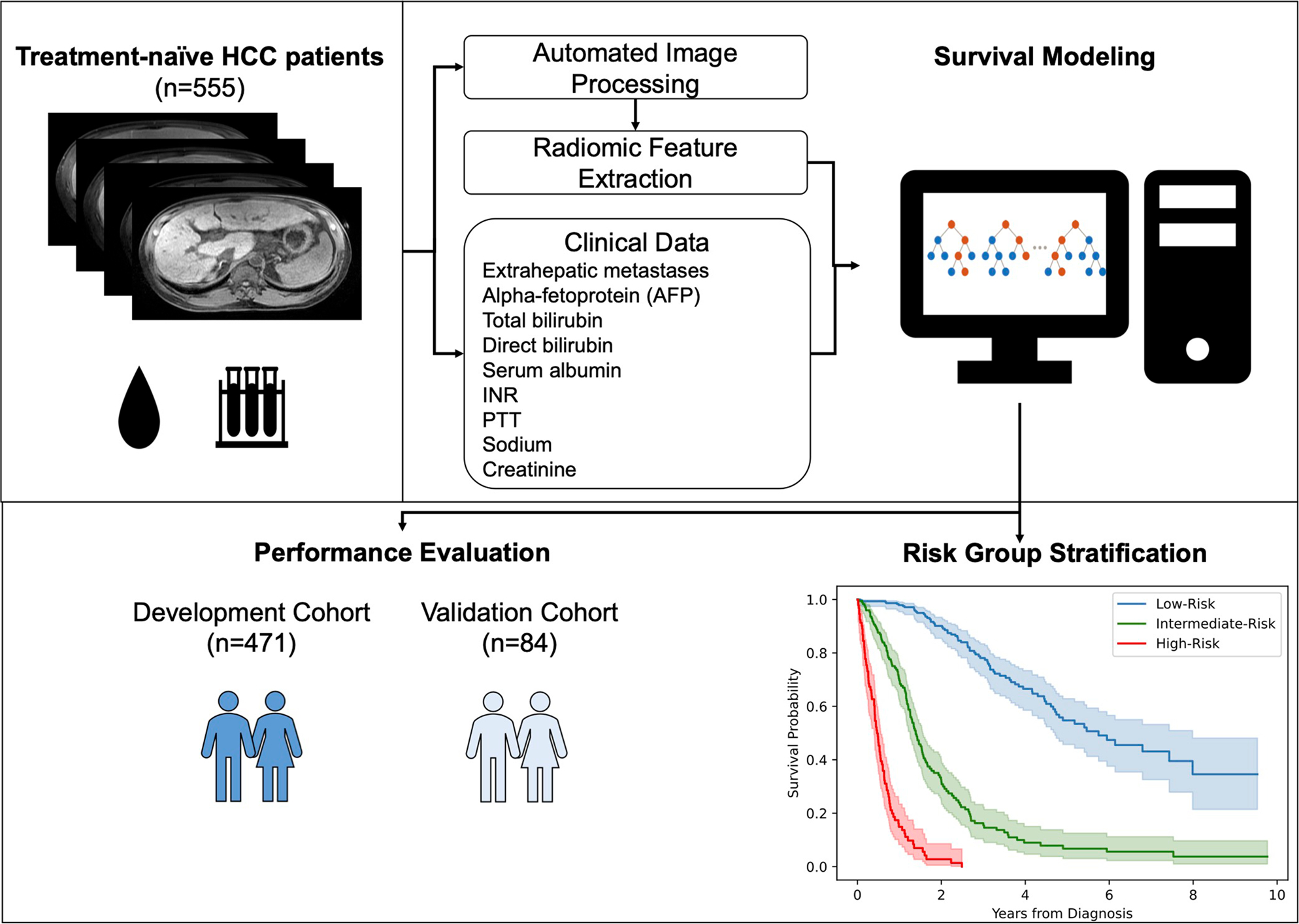
Model development. An automated liver segmentation framework was adopted for radiomic feature extraction after automated image co-registration. To predict overall survival, a random survival forest was fit from a combination of clinical and radiomic variables. Model performance was evaluated using Harrell’s C-index and the area under the time-dependent receiver operating characteristic curve (AUC). Patients were stratified into low-, intermediate-, and high-risk groups based on their predicted risk scores

**Fig. 3:**
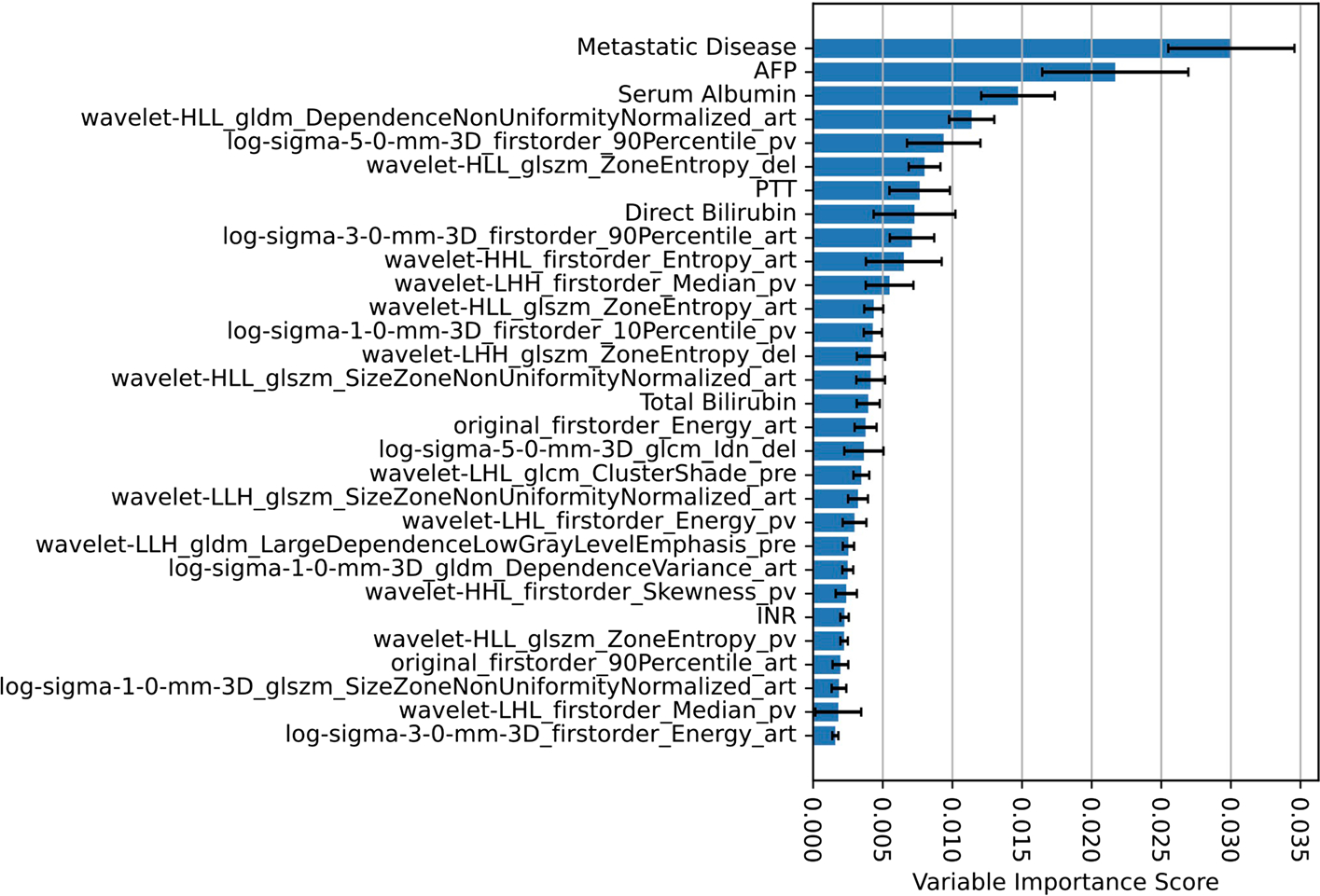
Variable importance scores. The bar chart shows the mean variable importance score (error bars show standard deviation) of each included variable of the final risk prediction model obtained by 10 permutations of random shuffling. Naming convention of radiomic features: The prefix specifies the image type (original image or filter-derived MR image (“log”: Laplacian of Gaussian) with extraction parameters); the suffix specifies the MR contrast phase (“_pre”: pre-contrast phase, “_art”: late arterial phase, “_pv”: portal venous phase, “_del”: delayed phase). Equations for the calculation of each radiomic feature are available in ref. [22]. (AFP: Alpha-fetoprotein; INR: international normalized ratio; PTT: partial thromboplastin time)

Figure 2 summarizes the entire model development pipeline. The proposed model attained C-indices of 0.8503 and 0.8234 in the development- and validation cohort, respectively. Table 2 summarizes all performance metrics for the proposed model and conventional clinical staging systems. For the interpretability of the proposed model, Fig. 3 depicts the variable importance scores of the included variables. On average, the proposed framework required a running time of 1.11 min per patient (automated liver segmentation, 0.70 s; extraction of 23 included radiomic features, 1.09 min; model prediction, 0.42 s).

### Mortality risk predictions and graded prognostic assessment

**Fig. 4:**
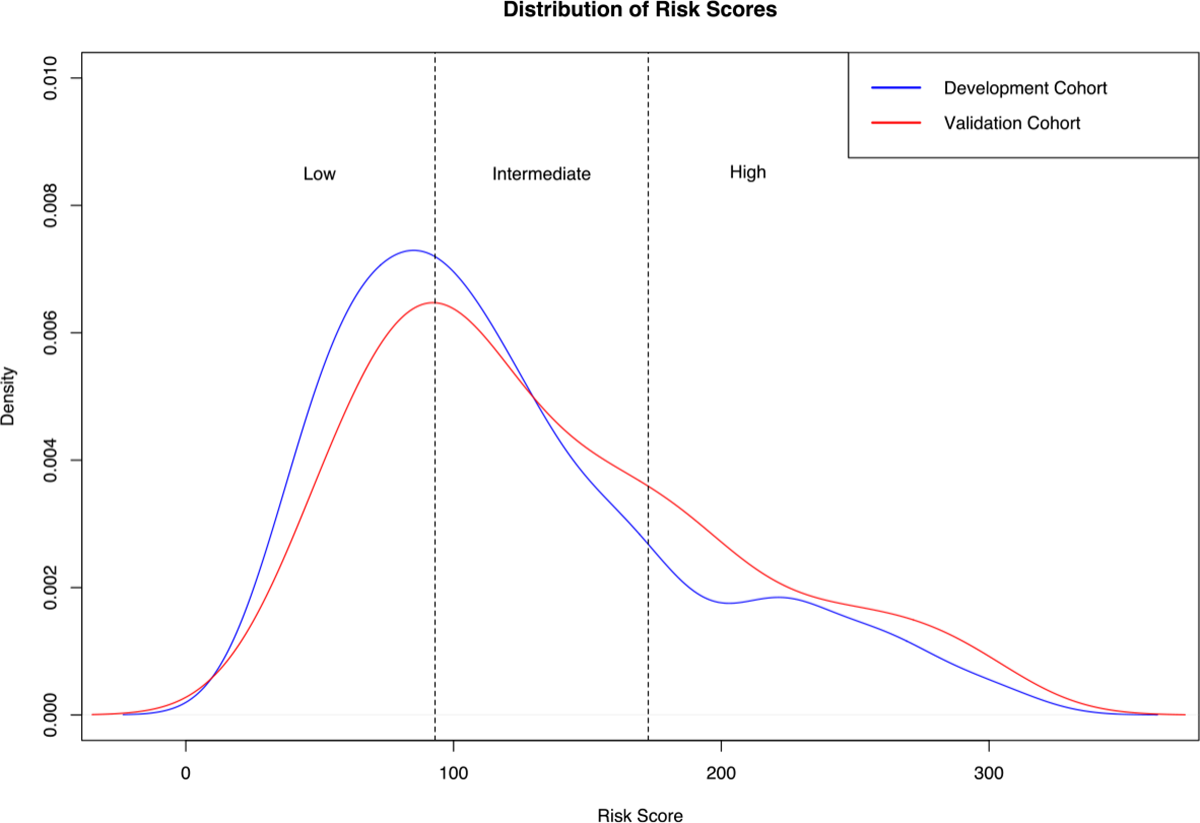
Distribution of predicted risk scores in the development- and independent validation cohort. Based on the risk score predictions of the survival model in the development cohort, we derived two cutoff points (93.08 and 172.73) to stratify patients into low-, intermediate-, and high-risk groups. We applied the same cutoff points for stratification in the independent validation cohort. For plotting, a Gaussian smoothing kernel was used

**Fig. 5:**
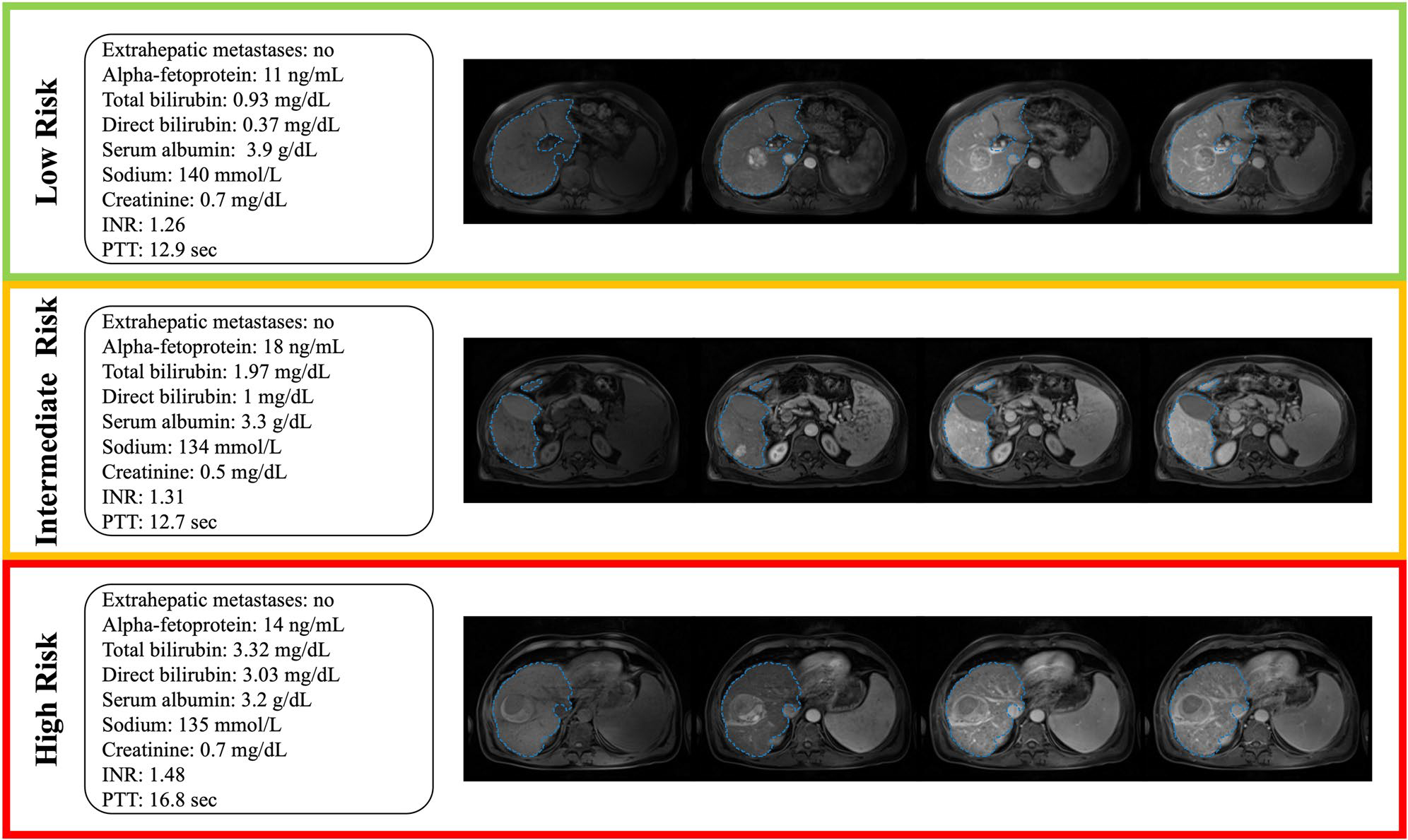
Example cases. Standard-of-care clinical data and axial pre-contrast-, late arterial, portal venous-, and delayed-phase MRI with corresponding automated liver segmentations overlaid in blue. Low-risk group: A 59-year-old male patient presenting with a focal 4.5 cm lesion in the right liver lobe. The patient was censored after 8.75 years. Intermediate-risk group: A 54-year-old male patient presenting with a focal 3.1 cm lesion in the right liver lobe. The patient died 15 months after diagnosis. High-risk group: A 54-year-old male patient presenting with a focal 5.7 cm lesion in the right liver lobe. The patient died 6.7 months after diagnosis

The distribution of risk scores in the development and validation cohort is shown in Fig. 4. In the development- and validation cohort, the mean (± standard deviation) predicted risk score was 121.57 (± 65.31) and 135.64 (± 67.59), respectively. Cox proportional hazards regression analysis showed a highly significant association between the predicted risk score and OS in the development cohort (coefficient, 0.021658 (*p* < .00001); HR, 1.022 (95% CI: 1.02, 1.024)), and in the validation cohort (coefficient, 0.021676 (*p*<*.00001*); HR, 1.022 (95% CI 1.016, 1.028)). The cutoff values determined by the hierarchical method to stratify patients into low-, intermediate-, and high-risk groups based on the proposed model’s predicted risk scores were 93.08 and 172.73. Detailed results of the Cox proportional hazards regression analysis for each risk group can be found in Supplemental Table 2. Example cases are shown in Fig. 5. In the development cohort, 193 (41%) patients were assigned to the low-risk group, 185 (39%) patients to the intermediate-risk group, and 93 (20%) patients to the high-risk group. In the validation cohort, 27 (32%) patients were allocated to the low-risk group, 32 (38%) patients to the intermediate-risk group, and 25 (30%) patients to the high-risk group. Supplemental Table 3 shows a cross-tabulation analysis of the proposed risk groups across conventional clinical staging systems.

### Survival analysis

**Fig. 6:**
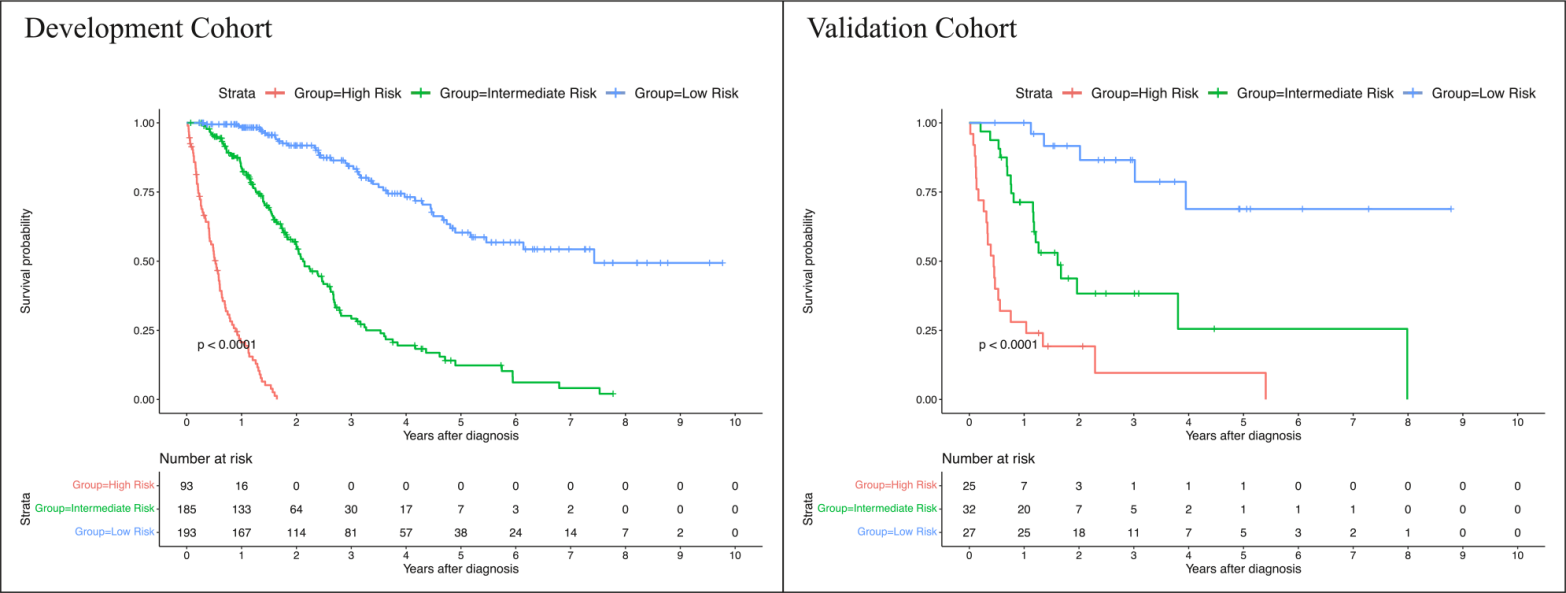
Kaplan-Meier curves of the proposed risk groups in the development- and validation cohort

A comprehensive table with mOS times and 95% confidence intervals for various strata can be found in Table 3. The mOS time of the entire study cohort was 32.47 (95% CI: 28.40, 37.83) months. In the development- and validation cohort, the mOS were 32.57 (95% CI: 29.13, 38.50) months and 24.57 (95% CI: 14.73, NA) months, respectively. Survival rates (± standard error) for the proposed risk groups in the development- and validation cohort are summarized in Table 4. Survival times between the development- and the validation cohort showed no statistical difference (*p* = .29). In the development cohort, mOS in the low-risk group was 90.40 (95% CI: 62.97, NA) months, 25.8 (95% CI: 23.50, 31.90) months in the intermediate-risk, and 6.40 (95% CI: 5.03, 7.73) months in the high-risk group. In the validation cohort, mOS in the low-risk group was NA (95% CI:48.03, NA) months, 19.60 (95% CI: 14.23, NA) months in the intermediate-risk, and 5.40 (95% CI: 3.80, 12.63) months in the high-risk group. Kaplan-Meier curves for the developed risk groups can be found in Fig. 6. The developed low-, intermediate-, and high-risk groups demonstrated significantly different survival times in both cohorts (development cohort, *p* <*.0001*; validation cohort, *p* <*.0001*). Notably, no statistical difference was found when comparing the survival times of each risk group between the development- and the validation cohort (low-risk group, *p* = 1.0; intermediate-risk group, *p* = 1.0; high-risk group, *p* = 1.0), thus indicating the generalizability of the risk groups’ survival times to new data. Complete results of the logrank test for pairwise OS comparisons between the proposed risk groups can be found in Supplemental Table 4. Kaplan-Meier curves for the conventional staging scores can be found in Supplemental Figure 1.

## Discussion

Using a large dataset (*n* = 555), we devised and independently validated a fully automated framework for mortality risk prediction in hepatocellular carcinoma patients using routinely available standard-of-care clinical data and radiomic biomarkers from automated liver segmentations on baseline multiphasic contrast-enhanced MRI. This completely data-driven method yielded reliable, fast, and reproducible risk predictions and attained state-of-the-art performances for mortality risk quantification at baseline. The generalizability of our method was confirmed in an independent validation cohort, and performance was compared against conventional staging systems. In addition, the proposed stratification into low-, intermediate- and high-risk groups yielded similar overall survival times in the development and validation cohort, indicating the generalizability of the method. By using automated liver segmentations, we ensure that the extracted radiomic imaging markers are stable and reproducible and increase the workflow substantially by demonstrating segmentation computation times under a second without the need for human interaction [17]. Finally, we developed our method using a data-driven approach. Instead of stratifying patients into risk groups based on one-dimensional image measurements [9–13], we derived risk group cutoff values using the full three-dimensional imaging volume itself. Thus, such a risk prediction framework could enable personalized follow-up strategies, guide management decisions, and improve clinical workflow efficiency in tumor boards.

We hypothesize that the proposed framework outperformed conventional systems for mortality risk quantification in terms of C-index and AUC as it uses advanced quantitative imaging biomarkers from the whole liver volume in imaging and a data-driven approach for mortality risk prediction. Three-dimensional quantitative assessment of tumor burden has been shown to be a stronger predictor of patient survival than one-dimensional tumor size measurements [23], and one-dimensional tumor size measurements, as used in conventional staging systems, have shown major limitations in reflecting viability, actual tumor size, and growth potential [24]. Our approach uses the full 3D MR imaging data for the extraction of quantitative biomarkers, which is a rich representation of MRI compared to single-dimensional representations such as diameter-based measurements. The 30 biomarkers with the highest importance scores derived from the data demonstrate a combination of clinical features and radiomics across all four phases of the MRI study (Fig. 4). These biomarkers effectively represent disease through intensity patterns and textures summarized by the radiomics in different phases of the imaging study. The performances of the conventional staging systems are in line with previously published studies [25–31]. Previous works have developed machine learning models for mortality risk quantification. However, direct comparison is challenging due to the different datasets, imaging modalities, and study endpoints being used. Mei et al. [32] developed and validated a prognostic nomogram to predict survival in patients with unresectable HCC after hepatic arterial infusion chemotherapy in a cohort with 463 predominantly advanced-stage patients attaining C-indices of 0.710 and 0.716 in their development and validation cohorts, respectively. Furthermore, the authors proposed a risk stratification approach to classify patients into three or four risk groups based on a trisection cutoff and a quartile cutoff point with significantly different OS times. While their approach did not include quantitative imagining biomarkers, their data-driven design achieved superior performance compared to conventional staging systems in their cohort, similar to the findings of our study. Liu et al. [33] developed and validated a prognostic nomogram to predict overall survival in HCC patients after hepatectomy from radiomic features from tumor segmentations on portal venous phase computed tomography and clinical- and pathological variables in a cohort with 544 Chinese patients and achieved C-indices of 0.747 and 0.777 in their development and validation cohorts, respectively. Based on the predicted risk scores, patients were allocated into low and high-risk groups with significantly different OS times. The authors could significantly improve performance when integrating radiomic features into the risk prediction model compared to clinical- and pathological features alone. Blanc-Durand et al. [34] proposed a scoring system to stratify HCC patients undergoing transarterial radioembolization with Yttrium-90 into a low- and a high-risk group. Radiomic features were derived from semi-automatic liver segmentations from pretreatment ^18^F-fluorodeoxyglucose positron emission tomography images. Their proposed risk score was significantly correlated with OS and could stratify patients into two risk groups with distinct OS times. However, their method was not evaluated on an independent validation cohort, and no measures of prognostic power, such as the C-index or time-dependent AUCs, were reported, and their method relies on manually revised semi-automatic liver segmentations. In their multi-institutional study, Ji et al. [35] presented a machine learning framework to predict the time to recurrence after resection. Their method was developed and evaluated in 470 patients with solitary HCC lesions and used radiomic features derived from manual tumor and peritumoral segmentations from baseline contrast-enhanced computed tomography images combined with clinical variables and achieved C-indices of 0.733–0.801, outperforming conventional staging systems. The authors proposed a risk stratification approach to allocate patients into low, intermediate, and high-risk groups with significantly different OS times. Their study confirms the utility of a data-driven approach and the usage of advanced quantitative imaging biomarkers for time-to-event data. However, their method relies on time-consuming manual tumor segmentation and does not use a fully automated segmentation method as our liver segmentation approach, which yields segmentation in processing times of under a second (0.70 s).

Our study has several limitations. First, our method was developed using retrospective data from a single institution; thus, prospective multicenter studies and external validation are warranted before potential clinical translation. However, our method was developed using a large dataset and yielded precise and generalizable results in an independent validation cohort. Furthermore, the large cohort size of this study adds a degree of robustness to the findings, and the relative simplicity of the analytical elements involved enhances the likelihood of reproducibility and the reliability of the findings. Due to these factors, we anticipate that other groups can replicate our study using the publicly available code. Second, we did not control for the effects of different treatment types on OS. Nevertheless, our method is still predictive of OS even without any treatment information given. Finally, the proposed method relies on multiphasic contrast-enhanced MRI and cannot be applied to patients who underwent other imaging modalities at baseline. In future work, we will incorporate longitudinal data from multiple external contributors and evaluate our method on a larger independent validation cohort.

In conclusion, we present a fully automated framework for mortality risk prediction in hepatocellular carcinoma patients using routinely available standard-of-care clinical data and radiomic features derived from automated liver segmentations on baseline multiphasic contrast-enhanced MRI outperforming conventional staging systems for mortality risk quantification. The developed method, based on machine learning, can help personalize follow-up strategies, guide management decisions, and improve clinical workflow efficiency in tumor boards.

## Supplementary Material

Supplementary Information

## Figures and Tables

**Table 1:** Patient baseline characteristics

Characteristic	Overall	Development cohort	Validation cohort	*p* value

Number of patients	555	471	84	
Follow-up (months), median [IQR]	17.43 [25.17]	17.83 [25.28]	15.35 [23.98]	1.00^#^
Dead	287 (51.7)	241 (51.2)	46 (54.8)	1.00^‡^
Demographics				
Age, mean (SD)	63.8 (8.9)	63.9 (9.0)	63.0 (8.5)	1.00^†^
Sex				
Female	118 (21.3)	101 (21.4)	17 (20.2)	1.00^‡^
Male	437 (78.7)	370 (78.6)	67 (79.8)	
Ethnicity*				
Asian	15 (2.7)	15 (3.2)	0 (0.0)	1.00^‡^
Black	72 (13.0)	61 (13.0)	11 (13.1)	
Hispanic	78 (14.1)	69 (14.6)	9 (10.7)	
Other/unknown	6 (1.1)	5 (1.1)	1 (1.2)	
White	384 (69.2)	321 (68.2)	63 (75.0)	
Cirrhosis	526 (94.8)	444 (94.3)	82 (97.6)	1.00^‡^
Etiology				
HCV*	319 (57.5)	271 (57.5)	48 (57.1)	1.00^‡^
HBV*	29 (5.2)	26 (5.5)	3 (3.6)	1.00^‡^
Alcohol	167 (30.1)	136 (28.9)	31 (36.9)	1.00^‡^
NAFLD*	87 (15.7)	77 (16.3)	10 (11.9)	1.00^‡^
Autoimmune	8 (1.4)	8 (1.7)	0 (0.0)	1.00^‡^
Cryptogenic	13 (2.3)	11 (2.3)	2 (2.4)	1.00^‡^
Not available	18 (3.2)	16 (3.4)	2 (2.4)	1.00^‡^
ECOG performance status				
0	405 (73.0)	341 (72.4)	64 (76.2)	1.00^‡^
1	106 (19.1)	94 (20.0)	12 (14.3)	
2	24 (4.3)	21 (4.5)	3 (3.6)	
3	10 (1.8)	7 (1.5)	3 (3.6)	
4	10 (1.8)	8 (1.7)	2 (2.4)	
Radiological data				
Number of lesions, mean (SD)	1.5 (1.1)	1.5 (1.0)	1.8 (1.6)	1.00^†^
Maximum tumor diameter (cm), mean (SD)	4.0 (3.2)	4.0 (3.2)	4.3 (3.4)	1.00^†^
Cumulative tumor diameter (cm), mean (SD)	4.6 (3.7)	4.6 (3.7)	4.9 (3.8)	1.00^†^
Liver lobe				
Bilobar	124 (22.3)	99 (21.0)	25 (29.8)	1.00^‡^
Left	110 (19.8)	95 (20.2)	15 (17.9)	
Right	321 (57.8)	277 (58.8)	44 (52.4)	
Ascites on imaging				
Absent	421 (75.9)	360 (76.4)	61 (72.6)	1.00^‡^
Slight	87 (15.7)	74 (15.7)	13 (15.5)	
Moderate	47 (8.5)	37 (7.9)	10 (11.9)	
Portal vein thrombosis	63 (11.4)	51 (10.8)	12 (14.3)	1.00^‡^
Tumor thrombus	51 (9.2)	41 (8.7)	10 (11.9)	1.00^‡^
Infiltrative disease	28 (5.0)	22 (4.7)	6 (7.1)	1.00^‡^
Metastatic disease	37 (6.7)	27 (5.7)	10 (11.9)	1.00^‡^
Laboratory values				
Alpha-Fetoprotein (ng/mL), mean (SD)	3799.0 (33,226.9)	2864.9 (18,534.2)	9037.1 (73,424.4)	1.00^†^
Total bilirubin (mg/dL), mean (SD)	1.4 (1.8)	1.4 (1.8)	1.5 (2.3)	1.00^†^
Direct bilirubin (mg/dL), mean (SD)	0.7 (1.3)	0.7 (1.3)	0.8 (1.3)	1.00^†^
Serum albumin (g/dL), mean (SD)	3.6 (0.6)	3.6 (0.6)	3.5 (0.6)	1.00^†^
Sodium (mmol/L), mean (SD)	137.9 (3.7)	138.0 (3.6)	137.3 (4.1)	1.00^†^
Creatinine (mg/dL), mean (SD)	1.0 (0.4)	1.0 (0.4)	1.0 (0.4)	1.00^†^
International normalized ratio, mean (SD)	1.2 (0.3)	1.2 (0.3)	1.2 (0.3)	1.00^†^
Partial thromboplastin time (sec), mean (SD)	12.7 (5.3)	12.7 (5.6)	12.6 (2.9)	1.00^†^
Staging systems				
Child-Pugh class				
A	336 (60.5)	288 (61.1)	48 (57.1)	1.00^‡^
B	184 (33.2)	156 (33.1)	28 (33.3)	
C	35 (6.3)	27 (5.7)	8 (9.5)	
BCLC* stage				
0	45 (8.1)	40 (8.5)	5 (6.0)	1.00^‡^
A	345 (62.2)	300 (63.7)	45 (53.6)	
B	70 (12.6)	57 (12.1)	13 (15.5)	
C	52 (9.4)	41 (8.7)	11 (13.1)	
D	43 (7.7)	33 (7.0)	10 (11.9)	
First-line treatments				
Transarterial chemoembolization	192 (34.6)	159 (33.8)	33 (39.3)	1.00^‡^
Ablation	138 (24.9)	113 (24.0)	25 (29.8)	
Liver resection	82 (14.8)	75 (15.9)	7 (8.3)	
Combined transarterial chemoembolization + ablation	68 (12.3)	63 (13.4)	5 (6.0)	
Sorafenib	24 (4.3)	19 (4.0)	5 (6.0)	
Best supportive care	24 (4.3)	18 (3.8)	6 (7.1)	
Y90-radioembolization	20 (3.6)	17 (3.6)	3 (3.6)	
Liver transplantation	7 (1.3)	7 (1.5)	0 (0)	

Numbers in parentheses are percentages. Ethnicity is provided through the electronic health record. To assess data consistency, the datasets were compared using a ^†^two sample t-test for continuous, a ^‡^Chi-squared test for categorical characteristics, and a ^#^logrank test for time-to-event data. *HCV*, hepatitis C virus; *HBV*, hepatitis B virus; NAFLD, non-alcoholic fatty liver disease; Child-Pugh [8]; *BCLC*, Barcelona Clinic Liver Cancer [9]

**Table 2:** Performance evaluation

		Performance Measure					
	Staging System	C-index	Time-dependent AUC				
			1 year	2 years	3 years	4 years	5 years

Development Cohort (n=471)	Proposed method	0.850	0.907	0.904	0.910	0.890	0.895
	Child-Pugh	0.735	0.721	0.686	0.641	0.630	0.637
	BCLC	0.733	0.808	0.749	0.747	0.748	0.697
	HKLC	0.741	0.807	0.773	0.745	0.730	0.708
	AJCC-TNM	0.699	0.770	0.717	0.707	0.724	0.665
	LCSGJ-TNM	0.697	0.763	0.714	0.712	0.720	0.668
	JIS	0.743	0.818	0.771	0.745	0.747	0.706
	ALBI-Grade	0.589	0.633	0.631	0.611	0.588	0.555

Validation Cohort (n=84)	Proposed method	0.823	0.893	0.867	0.860	0.790	0.765
	Child-Pugh	0.695	0.775	0.715	0.701	0.736	0.718
	BCLC	0.815	0.917	0.850	0.791	0.769	0.799
	HKLC	0.820	0.929	0.822	0.791	0.780	0.797
	AJCC-TNM	0.737	0.825	0.766	0.690	0.700	0.706
	LCSGJ-TNM	0.723	0.813	0.777	0.711	0.635	0.617
	JIS	0.813	0.922	0.855	0.796	0.756	0.734
	ALBI-Grade	0.625	0.635	0.693	0.706	0.701	0.679

To evaluate the performance of mortality risk stratification, we calculated Harrel’s C-index [19] and the area under the time-dependent receiver operating characteristic curve [20] (“AUC”). Child-Pugh [8]; *BCLC*, Barcelona Clinic Liver Cancer [9]; *HKLC*, Hong Kong Liver Cancer [10]; *AJCC*, American Joint Committee on Cancer 8^th^ edition [11]; *LCSGJ-TNM*, Liver Cancer Study Group of Japan Tumor Node Metastasis [12]; *JIS*, Japan Integrated Staging [13]; *ALBI*, albumin-bilirubin [14]

**Table 3:** Median overall survival times across staging systems

Strata	Median OS in months (95% CI)	
	Development cohort	Validation cohort

Entire cohort	32.57 (29.13, 38.5)	24.57 (14.73, NA)
Proposed risk groups		
Low risk	90.4 (62.97, NA)	NA (48.03, NA)
Intermediate risk	25.8 (23.5, 31.9)	19.6 (14.23, NA)
High Risk	6.4 (5, 7.8)	5.4 (3.8, NA)
Child-Pugh		
A	43.6 (36.97, 56.77)	97.17 (24.57, NA)
B	18.6 (15.9, 26.13)	15.37 (6.8, NA)
C	4.83 (2.1, 5.9)	3.92 (1.23, NA)
BCLC stage		
0	74.7 (57.67, NA)	NA (NA, NA)
A	39.8 (32.93, 53.1)	65.77 (36.73, NA)
B	18.33 (16.43, 25.8)	13.67 (9.3, NA)
C	7.73 (5.83, 10.43)	5.4 (4.1, NA)
D	3.5 (2.1, 5.1)	2.65 (1.23, NA)
HKLC stage		
I	57.67 (52.3, 91.6)	NA (36.73, NA)
II	28.63 (24.33, 33.87)	46.3 (23.9, NA)
III	16.93 (15.23, 21.47)	8 (6.5, NA)
IV	9.07 (6.4, NA)	5.47 (4.77, NA)
V	3.27 (2.5, 5.1)	2.65 (1.23, NA)
AJCC-TNM		
IA	57.67 (50.7, 74.7)	65.77 (24.57, NA)
IB	38.5 (30.3, 54.1)	46.3 (14.73, NA)
II	25.23 (21, 35.17)	9.3 (6.5, NA)
IIIA	17.8 (15.83, 44.53)	3.8 (1.6, NA)
IIIB	5.83 (3.7, 16.93)	1.13 (0.23, NA)
IVA	7.97 (1.6, NA)	5.47 (4.77, NA)
IVB	2.67 (2.1, 7.1)	48.03 (20.33, NA)
LCSGJ-TNM		
I	57.67 (50.7, 74.7)	48.03 (36.73, NA)
II	39.8 (32.57, 54.3)	9.83 (8.33, NA)
III	21 (17.4, 29.13)	2.03 (0.23, NA)
IVA	6.78 (3.7, 19.73)	5.47 (4.77, NA)
IVB	2.67 (2.1, 7.1)	NA (27.9, NA)
JIS		
0	59.6 (54.7, NA)	NA (NA, NA)
1	45.7 (32.93, 62.97)	48.03 (27.9, NA)
2	30.3 (21,42.57)	20.33 (14.73, NA)
3	14.67 (8.53, 19.5)	9.2 (2.03, NA)
4	3.5 (2.23, 7.1)	4.07 (3.8, NA)
5	1.6 (0.5, NA)	0.73 (0.23, NA)
ALBI grade		
1	39.43 (33.87, 53.1)	2.5 (NA, NA)
2	12.13 (3.27, NA)	14.73 (5.7, NA)
3	22.1 (16.67, 27.43)	NA (NA, NA)

NA values indicate that more than 50% of the population was still alive at the end of the analysis; thus, median OS times cannot be calculated. *Child-Pugh* [8]; *BCLC*, Barcelona Clinic Liver Cancer [9]; *HKLC*, Hong Kong Liver Cancer [10]; *AJCC*, American Joint Committee on Cancer 8^th^ edition [11]; *LCSGJ-TNM*, Liver Cancer Study Group of Japan Tumor Node Metastasis [12]; *JIS*, Japan Integrated Staging [13]; *ALBI*, albumin-bilirubin [14]

**Table 4:** Survival rates (± standard error) for the proposed risk groups

Proposed risk group	Years	Survival rates (± Standard error)	
		Development cohort (*n* = 471)	Validation cohort (*n* = 84)

Low risk	1	0.98 (± 0.01)	1.00 (± 0.00)
	3	0.85 (± 0.03)	0.85 (± 0.08)
	5	0.61 (± 0.05)	0.68 (± 0.13)
Intermediate risk	1	0.83 (± 0.03)	0.70 (± 0.08)
	3	0.31 (± 0.04)	0.38 (± 0.10)
	5	0.13 (± 0.04)	0.29 (± 0.11)
High risk	1	0.23 (± 0.05)	0.28 (± 0.09)
	3	0.00 (± 0.00)	0.11 (± 0.08)
	5	0.00 (± 0.00)	0.11 (± 0.08)

## Abbreviations

AFP: Alpha-fetoprotein
AUC: Area under the curve
CI: Confidence interval
CT: Computed tomography
HCC: Hepatocellular carcinoma
INR: International normalized ratio
IQR: Interquartile range
mOS: Median overall survival
MRI: Magnetic resonance imaging
NA: Not available
PTT: Partial thromboplastin time
RSF: Random survival forest
SD: Standard deviation
