# Supplementary material for: Automated graded prognostic assessment for patients with hepatocellular carcinoma using machine learning
Source: Eur Radiol. Author manuscript; Available in PMC 2024 Oct 1. (PMC11399284; doi:10.1007/s00330-024-10624-8)
Supplement: Supplementary Information [file NIHMS2006482-supplement-Supplementary_Information.docx]

# Supplemental Material

for the original article

**Automated Graded Prognostic Assessment for Patients with Hepatocellular Carcinoma Using Machine Learning**

**Supplement 1. Image processing.**

***Automated image co-registration***

All images were converted to the Neuroimaging Informatics Technology Initiative (NIfTI) format using the software dcm2nii (v1.0.20210317) [1]. Pre-contrast-, late arterial-, and delayed-phase images were co-registered to the portal-venous phase images by applying a non-rigid intensity-based registration using the software BioImage Suite (v3.5) [2]. We parameterized the non-rigid transformation as a free-form deformation (FFD) using 3D B-splines [3]. We estimated the optimal FFD transformation by maximizing the normalized mutual information similarity metric [4] using gradient descent optimization. This optimization utilized a multi-resolution image pyramid with three levels with the final B-spline control point spacing set to 80 mm. The estimated transformation was then used to warp the moving image to the reference image space. We used this procedure to register the pre-contrast-, arterial-, and delayed phase image (moving images) to the portal venous image (reference image).

***Automated liver segmentation***

A fully convolutional neural network based on deep learning was used for automated whole liver segmentation [5] (available at https://github.com/OnofreyLab/volumetry-net/) and implemented using Python (v3.8.11), PyTorch (v1.5.1), and the open-source Medical Open Network for AI (MONAI) (v0.3.0) framework on a Linux workstation with an NVIDIA Quadro RTX 8000 GPU. The generated liver segmentations were saved in the NIfTI format for further processing.

***Radiomic feature extraction***

Radiomic features based on liver shape (n=14), intensity (n=18), and texture (n=75) were extracted from the automated liver segmentation masks using the software PyRadiomics (v3.0) [6]. All images were resized to isotropic 1.25x1.25x1.25 mm voxel spacing using B-spline interpolation. Subsequently, all MR images were normalized to have a mean signal intensity of 0 and a standard deviation of 100. For feature extraction, a fixed bin width of 25 was used. The intensity- and texture-based features were extracted from 12 different image types that were generated from the original image and 11 image derivatives by applying three edge-enhancement Laplacian of Gaussian filters (with sigma values of 1, 3, and 5 mm) and eight wavelet filters to the original image, creating 12 image types per MR contrast phase. This resulted in a total of 4,478 extracted features from the four MR contrast phases (4 MRI contrast phases x 12 image types x (18 intensity features + 75 texture features) + 14 shape features). The customized PyRadiomics settings are provided in **Supplement 2,** and a list of the extracted features can be found in **Supplemental Table 5**. The equations for the calculation of each feature are available in ref. [7].

**Supplement 2. PyRadiomics extraction parameters.**

Radiomic features were extracted from the automated liver segmentation masks using a customized PyRadiomics (v3.0) [6] pipeline. The used extraction parameters can be found below:

ImageType:

LoG:

sigma:

-1.0

-3.0

-5.0

Original:

Wavelet:

setting:

additionalInfo: true

binWidth: 25

interpolator: sitkBSpline

label: 1

normalize: true

normalizeScale: 100

removeOutliers: 3

resampledPixelSpacing:

-1.25

-1.25

-1.25

**Supplemental Table 1. Magnetic resonance imaging parameters.**

| **Parameter** | **Overall** | **Development Cohort** | **Validation Cohort** |
| --- | --- | --- | --- |
| Number of Scans | 555 | 471 | 84 |
| Manufacturer | | | |
| GE Medical Systems | 103 (18.6) | 86 (18.3) | 17 (20.2) |
| Hitachi | 8 (1.4) | 5 (1.1) | 3 (3.6) |
| Philips | 7 (1.3) | 6 (1.3) | 1 (1.2) |
| Siemens | 431 (77.7) | 369 (78.3) | 62 (73.8) |
| Toshiba | 6 (1.1) | 5 (1.1) | 1 (1.2) |
| Model Name | | | |
| Achieva | 1 (0.2) | 1 (0.2) | 0 (0.0) |
| Aera | 82 (14.8) | 68 (14.4) | 14 (16.7) |
| Avanto | 93 (16.8) | 84 (17.8) | 9 (10.7) |
| Espree | 59 (10.6) | 49 (10.4) | 10 (11.9) |
| Ingenia | 6 (1.1) | 5 (1.1) | 1 (1.2) |
| MAGNETOM Vida | 1 (0.2) | 1 (0.2) | 0 (0.0) |
| MRT200SP8 | 4 (0.7) | 3 (0.6) | 1 (1.2) |
| OASIS | 8 (1.4) | 5 (1.1) | 3 (3.6) |
| Optima MR450w | 12 (2.2) | 9 (1.9) | 3 (3.6) |
| Signa | 1 (0.2) | 1 (0.2) | 0 (0.0) |
| Signa Excite | 44 (7.9) | 37 (7.9) | 7 (8.3) |
| Signa HDe | 1 (0.2) | 1 (0.2) | 0 (0.0) |
| Signa HDx | 21 (3.8) | 20 (4.2) | 1 (1.2) |
| Signa HDxt | 17 (3.1) | 14 (3.0) | 3 (3.6) |
| Skyra | 13 (2.3) | 13 (2.8) | 0 (0.0) |
| Symphony | 10 (1.8) | 10 (2.1) | 0 (0.0) |
| SymphonyTim | 1 (0.2) | 1 (0.2) | 0 (0.0) |
| TrioTim | 19 (3.4) | 16 (3.4) | 3 (3.6) |
| Verio | 147 (26.5) | 122 (25.9) | 25 (29.8) |
| not available | 15 (2.7) | 11 (2.3) | 4 (4.8) |
| Magnetic Field Strength (T) | | | |
| 1.16 | 8 (1.4) | 5 (1.1) | 3 (3.6) |
| 1.5 | 356 (64.1) | 303 (64.3) | 53 (63.1) |
| 3 | 191 (34.4) | 163 (34.6) | 28 (33.3) |
| Contrast Agent | | | |
| Dotarem | 122 (22.0) | 104 (22.1) | 18 (21.4) |
| Eovist | 28 (5.0) | 24 (5.1) | 4 (4.8) |
| Gadovist | 273 (49.2) | 229 (48.6) | 44 (52.4) |
| Magnevist | 97 (17.5) | 88 (18.7) | 9 (10.7) |
| MultiHance | 17 (3.1) | 14 (3.0) | 3 (3.6) |
| Omniscan | 7 (1.3) | 6 (1.3) | 1 (1.2) |
| Optimark | 7 (1.3) | 4 (0.8) | 3 (3.6) |
| ProHance | 4 (0.7) | 2 (0.4) | 2 (2.4) |
| Echo numbers, mean (SD) | 1.0 (0.1) | 1.0 (0.1) | 1.0 (0.0) |
| Echo time, mean (SD) | 1.9 (0.5) | 1.9 (0.5) | 1.9 (0.5) |
| Imaging frequency (MHz), mean (SD) | 2313230.8 (38393991.2) | 2722593.8 (41646292.7) | 83.3 (28.8) |
| Bandwidth (Hz), mean (SD) | 418.5 (125.5) | 421.1 (132.1) | 402.7 (74.1) |
| Repetition time (ms), mean (SD) | 5.6 (16.4) | 5.7 (17.8) | 4.5 (1.0) |
| Slice thickness (mm), mean (SD) | 3.5 (0.9) | 3.5 (0.9) | 3.7 (1.1) |
| Note. —Numbers in parentheses are percentages if not indicated otherwise. | | | |

**Supplemental Table 2. Cox proportional hazards regression analysis.**

| **Factor** | **Cohort** | **Coefficient** | ***P* value** | **HR (95% CI)** |
| --- | --- | --- | --- | --- |
| Risk score | Development cohort | 0.0217 | *<.0001* | 1.0219 (1.0200, 1.0240) |
|  | Validation cohort | 0.0217 | *<.0001* | 1.0219 (1.0160, 1.0280) |
| Risk groups | Development cohort | | | |
|  | Low Risk (reference) |  |  |  |
|  | Intermediate Risk | 1.689 | *<.0001* | 5.4150 (3.7800, 7.7590) |
|  | High Risk | 4.0247 | *<.0001* | 55.9650 (35.3900, 88.5030) |
|  | Validation cohort | | | |
|  | Low Risk (reference) |  |  |  |
|  | Intermediate Risk | 1.5958 | *.0017* | 4.9320 (1.8240, 13.3400) |
|  | High Risk | 2.6910 | *<.0001* | 14.7467 (5.4780, 39.7000) |

**Supplemental Table 3. Cross Tabulation Analysis.**

| **Staging System** | **Proposed Risk Groups** | | | | | |
| --- | --- | --- | --- | --- | --- | --- |
|  | **Development Cohort (n=471)** | | | **Evaluation Cohort (n=84)** | | |
|  | **Low** | **Intermediate** | **High** | **Low** | **Intermediate** | **High** |
| **Child-Pugh** | | | | | | |
| A | 162 | 110 | 16 | 25 | 18 | 5 |
| B | 31 | 73 | 52 | 2 | 14 | 12 |
| C | 0 | 3 | 24 | 0 | 0 | 8 |
| **BCLC** | | | | | | |
| 0 | 30 | 10 | 0 | 4 | 1 | 0 |
| A | 141 | 133 | 26 | 18 | 20 | 7 |
| B | 17 | 30 | 11 | 5 | 8 | 0 |
| C | 5 | 9 | 26 | 0 | 3 | 8 |
| D | 0 | 4 | 29 | 0 | 0 | 10 |
| **HKLC** | | | | | | |
| I | 125 | 61 | 3 | 19 | 11 | 2 |
| II | 62 | 98 | 27 | 7 | 15 | 4 |
| III | 4 | 19 | 11 | 1 | 4 | 3 |
| IV | 1 | 2 | 13 | 0 | 2 | 6 |
| V | 1 | 6 | 38 | 0 | 0 | 10 |
| **AJCC-TNM** | | | | | | |
| IA | 47 | 38 | 6 | 6 | 4 | 3 |
| IB | 89 | 89 | 20 | 11 | 12 | 5 |
| II | 47 | 44 | 19 | 9 | 10 | 4 |
| IIIA | 5 | 6 | 5 | 1 | 3 | 1 |
| IIIB | 5 | 7 | 18 | 0 | 1 | 4 |
| IVA | 0 | 1 | 2 | 0 | 0 | 2 |
| IVB | 0 | 1 | 22 | 0 | 2 | 6 |
| **LCSGJ-TNM** | | | | | | |
| I | 47 | 38 | 6 | 6 | 4 | 3 |
| II | 104 | 95 | 27 | 14 | 14 | 5 |
| III | 40 | 48 | 25 | 7 | 12 | 8 |
| IVA | 2 | 4 | 12 | 0 | 0 | 3 |
| IVB | 0 | 1 | 22 | 0 | 2 | 6 |
| **JIS** | | | | | | |
| 0 | 40 | 16 | 0 | 6 | 1 | 0 |
| 1 | 92 | 88 | 8 | 12 | 10 | 3 |
| 2 | 54 | 50 | 20 | 9 | 15 | 5 |
| 3 | 7 | 31 | 32 | 0 | 6 | 7 |
| 4 | 0 | 1 | 25 | 0 | 0 | 8 |
| 5 | 0 | 0 | 7 | 0 | 0 | 2 |
| **ALBI Grade** | | | | | | |
| 1 | 150 | 102 | 23 | 26 | 19 | 5 |
| 2 | 1 | 1 | 1 | 0 | 1 | 0 |
| 3 | 42 | 83 | 68 | 1 | 12 | 20 |
| Note. —Values indicate the number of subjects in each category. Child-Pugh [8]; BCLC, Barcelona Clinic Liver Cancer [9]; HKLC, Hong Kong Liver Cancer [10]; AJCC, American Joint Committee on Cancer 8^th^ edition [11]; LCSGJ-TNM, Liver Cancer Study Group of Japan tumor node metastasis [12]; JIS, Japan Integrated Staging [13]; ALBI, Albumin-Bilirubin [14] | | | | | | |

**Supplemental Table 4. Logrank test risk group comparisons between the development and validation cohorts.**

|  | **High Risk**  **(Development)** | **High Risk**  **(Validation)** | **Intermediate Risk**  **(Development)** | **Intermediate Risk**  **(Validation)** | **Low Risk**  **(Development)** |
| --- | --- | --- | --- | --- | --- |
| **High Risk**  **(Validation)** | *1.0* |  |  |  |  |
| **Intermediate Risk**  **(Development)** | *<.00001* | *<.00001* |  |  |  |
| **Intermediate Risk**  **(Validation)** | *<.00001* | *.00572* | *1.0* |  |  |
| **Low Risk**  **(Development)** | *<.00001* | *<.00001* | *<.00001* | *<.00001* |  |
| **Low Risk**  **(Validation)** | *<.00001* | *<.00001* | *.00006* | *.00282* | *1.0* |

**Supplemental Table 5.** **List of extracted radiomic features.**

A complete list of PyRadiomics [6] features used in this study. The equations for the calculation of each feature are available in ref. [7]. Intensity- and texture-based features were derived from the original image, and after applying 8 wavelet and 3 Laplacian of Gaussian (LoG) filters, creating 12 image types per MR contrast phase. This resulted in a total of 4478 extracted features from the four MR contrast phases (4 MRI contrast phases x 12 image types x (18 intensity features + 75 texture features) + 14 shape features).

| Feature Family | | Feature Name |
| --- | --- | --- |
| Intensity: First-Order | 1 | 10th Percentile |
|  | 2 | 90th Percentile |
|  | 3 | Energy |
|  | 4 | Entropy |
|  | 5 | Interquartile Range |
|  | 6 | Kurtosis |
|  | 7 | Maximum |
|  | 8 | Mean Absolute Deviation |
|  | 9 | Mean |
|  | 10 | Median |
|  | 11 | Minimum |
|  | 12 | Range |
|  | 13 | Robust Mean Absolute Deviation |
|  | 14 | Root MeanSquared |
|  | 15 | Skewness |
|  | 16 | Total Energy |
|  | 17 | Uniformity |
|  | 18 | Variance |
| Texture: Gray-Level Co-Occurrence Matrix (glcm) | 1 | Autocorrelation |
|  | 2 | Cluster Prominence |
|  | 3 | Cluster Shade |
|  | 4 | Cluster Tendency |
|  | 5 | Contrast |
|  | 6 | Correlation |
|  | 7 | Difference Average |
|  | 8 | Difference Entropy |
|  | 9 | Difference Variance |
|  | 10 | Inverse Difference |
|  | 11 | Inverse Difference Moment |
|  | 12 | Inverse Difference Moment Normalized |
|  | 13 | Inverse Difference Normalized |
|  | 14 | Informational Measure of Correlation 1 |
|  | 15 | Informational Measure of Correlation 2 |
|  | 16 | Inverse Variance |
|  | 17 | Joint Average |
|  | 18 | Joint Energy |
|  | 19 | Joint Entropy |
|  | 20 | Maximal Correlation Coefficient |
|  | 21 | Maximum Probability |
|  | 22 | Sum Average |
|  | 23 | Sum Entropy |
|  | 24 | Sum Squares |
| Texture: Gray Level Dependence Matrix (gldm) | 1 | Dependence Entropy |
|  | 2 | Dependence Non-Uniformity |
|  | 3 | Dependence Non-Uniformity Normalized |
|  | 4 | Dependence Variance |
|  | 5 | Gray Level Non-Uniformity |
|  | 6 | Gray Level Variance |
|  | 7 | High Gray Level Emphasis |
|  | 8 | Large Dependence Emphasis |
|  | 9 | Large Dependence High Gray Level Emphasis |
|  | 10 | Large Dependence Low Gray Level Emphasis |
|  | 11 | Low Gray Level Emphasis |
|  | 12 | Small Dependence Emphasis |
|  | 13 | Small Dependence High Gray Level Emphasis |
|  | 14 | Small Dependence Low Gray Level Emphasis |
| Texture: Gray Level Run Length Matrix (glrlm) | 1 | Gray Level Non-Uniformity |
|  | 2 | Gray Level Non-Uniformity Normalized |
|  | 3 | Gray Level Variance |
|  | 4 | High Gray Level Run Emphasis |
|  | 5 | Long Run Emphasis |
|  | 6 | Long Run High Gray Level Emphasis |
|  | 7 | Long Run Low Gray Level Emphasis |
|  | 8 | Low Gray Level Run Emphasis |
|  | 9 | Run Entropy |
|  | 10 | Run Length Non-Uniformity |
|  | 11 | Run Length Non-Uniformity Normalized |
|  | 12 | Run Percentage |
|  | 13 | Run Variance |
|  | 14 | Short Run Emphasis |
|  | 15 | Short Run High Gray Level Emphasis |
|  | 16 | Short Run Low Gray Level Emphasis |
| Texture: Gray Level Size Zone Matrix (glszm) | 1 | Gray Level Non-Uniformity |
|  | 2 | Gray Level Non-Uniformity Normalized |
|  | 3 | Gray Level Variance |
|  | 4 | High Gray Level Zone Emphasis |
|  | 5 | Large Area Emphasis |
|  | 6 | Large Area High Gray Level Emphasis |
|  | 7 | Large Area Low Gray Level Emphasis |
|  | 8 | Low Gray Level Zone Emphasis |
|  | 9 | Size Zone Non-Uniformity |
|  | 10 | Size Zone Non-Uniformity Normalized |
|  | 11 | Small Area Emphasis |
|  | 12 | Small Area High Gray Level Emphasis |
|  | 13 | Small Area Low Gray Level Emphasis |
|  | 14 | Zone Entropy |
|  | 15 | Zone Percentage |
|  | 16 | Zone Variance |
| Texture: Neighboring Gray Tone Difference Matrix (ngtdm) | 1 | Busyness |
|  | 2 | Coarseness |
|  | 3 | Complexity |
|  | 4 | Contrast |
|  | 5 | Strength |
| Shape | 1 | Elongation |
|  | 2 | Flatness |
|  | 3 | Least Axis Length |
|  | 4 | Major Axis Length |
|  | 5 | Maximum 2D Diameter Axial |
|  | 6 | Maximum 2D Diameter Coronal |
|  | 7 | Maximum 2D Diameter Slice |
|  | 8 | Maximum 3D Diameter |
|  | 9 | Mesh Volume |
|  | 10 | Minor Axis Length |
|  | 11 | Sphericity |
|  | 12 | Surface Area |
|  | 13 | Surface Volume Ratio |
|  | 14 | Voxel Volume |

**Supplemental Figure 1. Kaplan-Meier Curves.** Kaplan Meier curves across both the development and the validation cohorts for the different conventional staging systems.

Figure S1.01 Kaplan Meier curve of the study population.

Figure S1.02 Development and validation cohorts.

Figure S1.03 First Treatments

*P <.0001*

Figure S1.04 Child-Pugh.


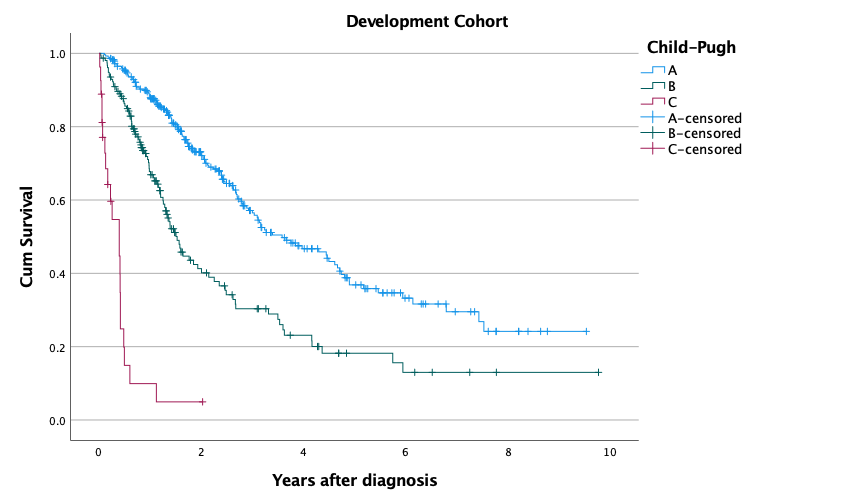


*P <.0001*


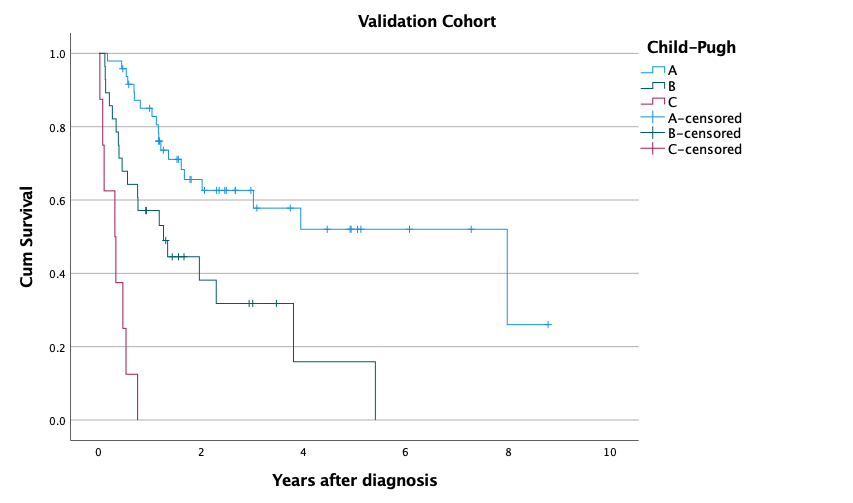


*P <.0001*

Figure S1.05 BCLC.


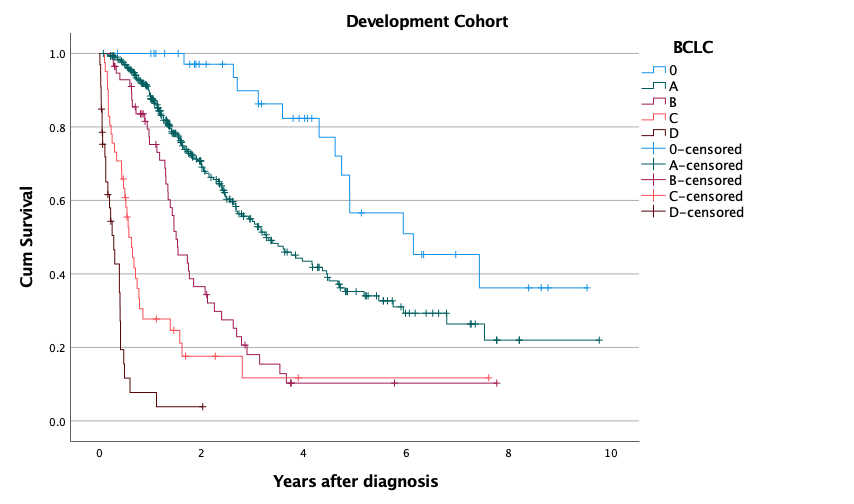


*P <.0001*


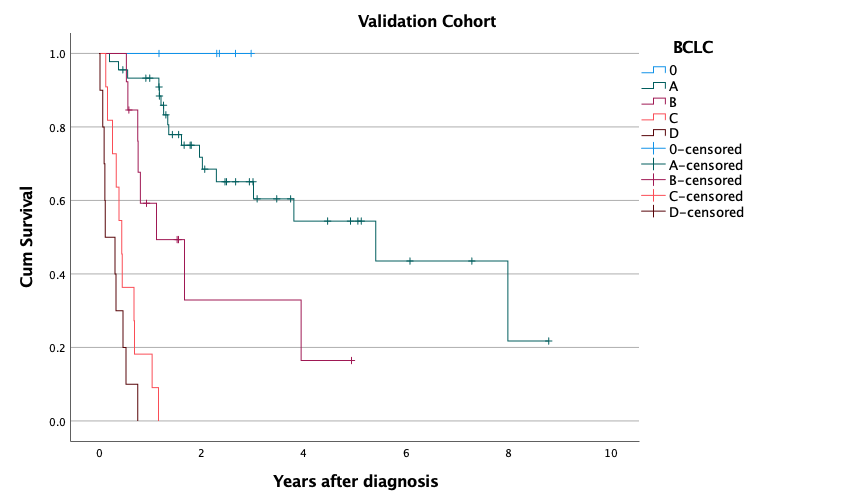


*P <.0001*

Figure S1.06 HKLC.


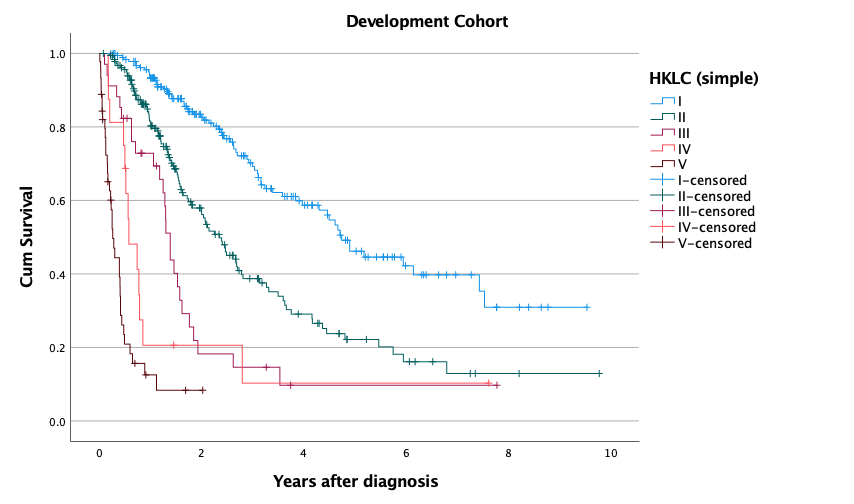


*P <.0001*


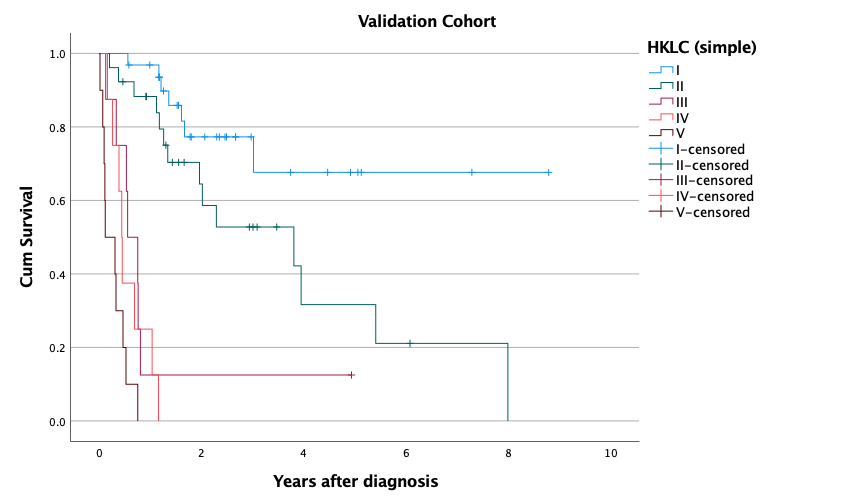


*P <.0001*

Figure S1.07 AJCC-TNM.


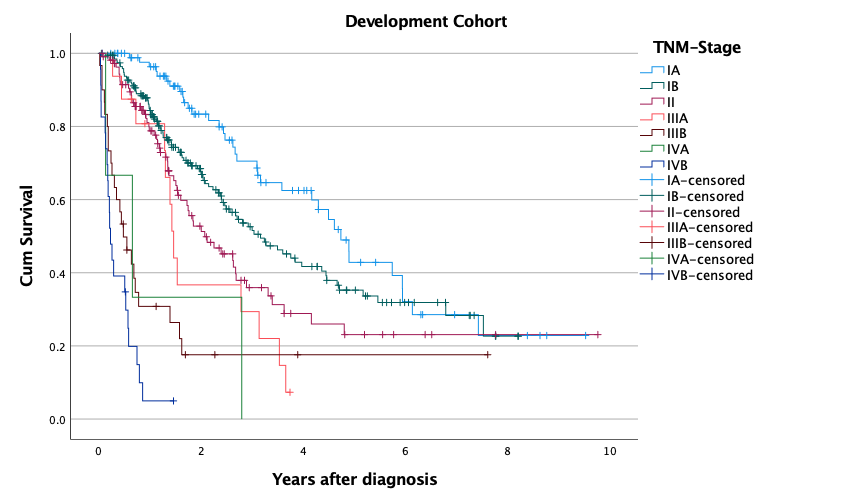


*P <.0001*


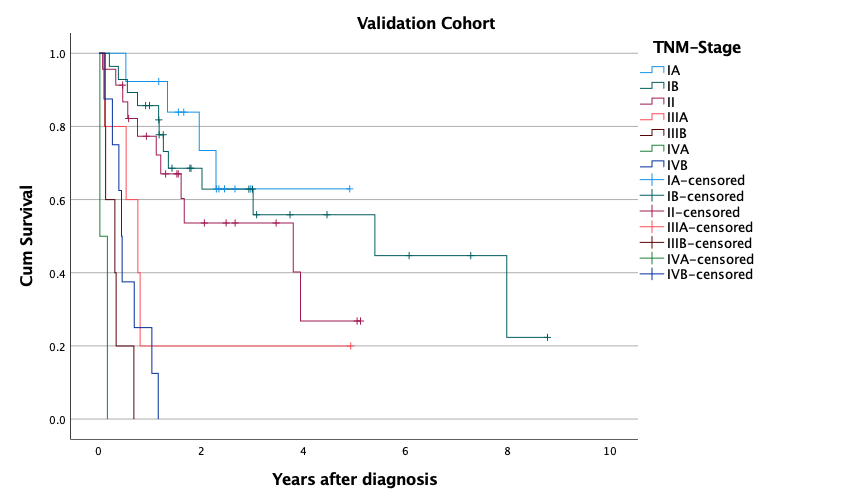


*P <.0001*

Figure S1.08 LCSGJ-TNM.


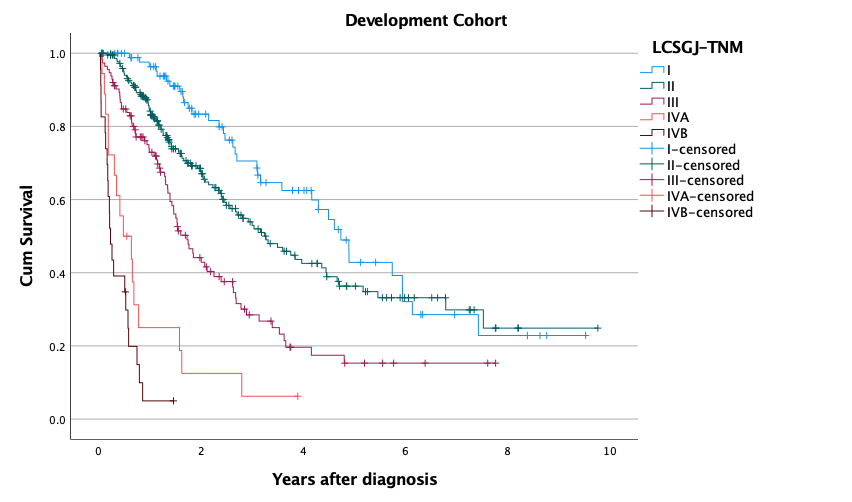


*P <.0001*


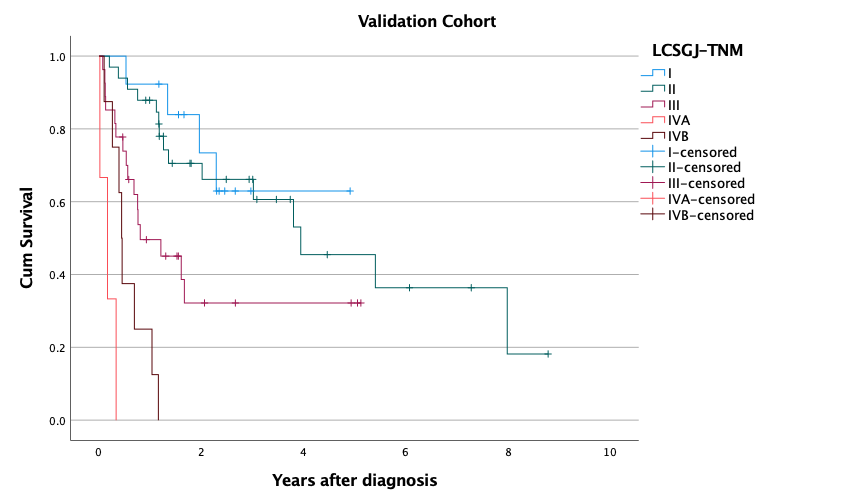


*P <.0001*

Figure S1.09 JIS.


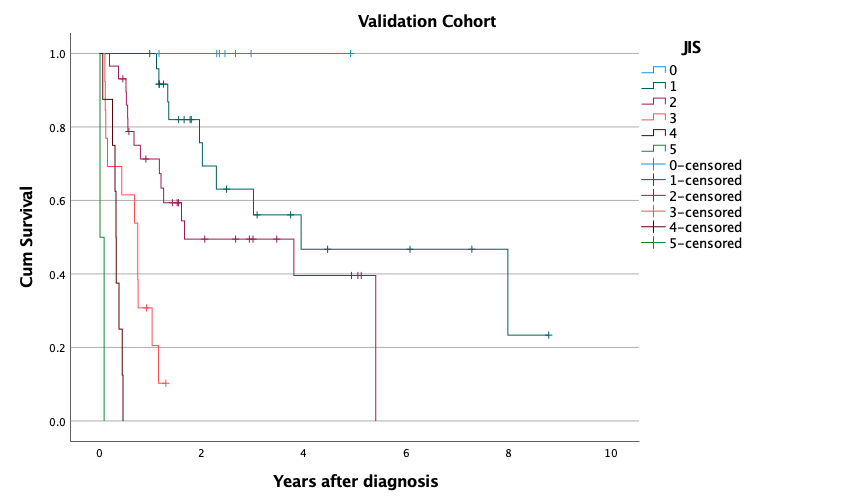


*P <.0001*


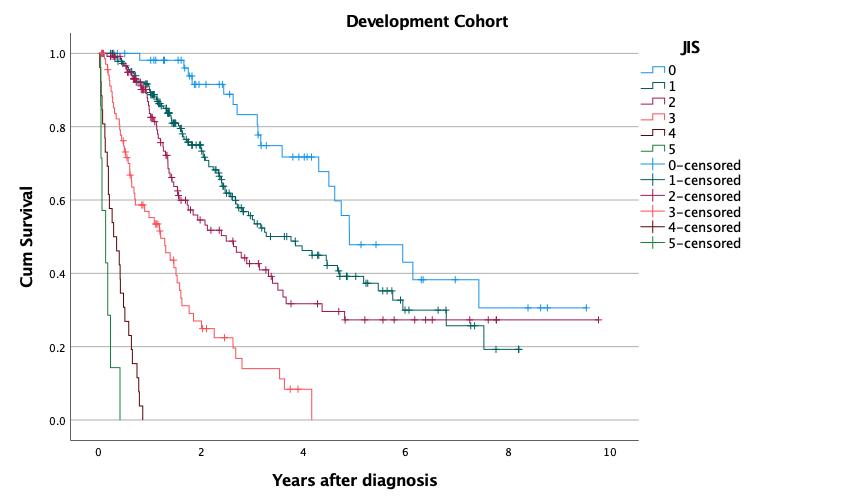


*P <.0001*

Figure S1.10 ALBI-Grade.


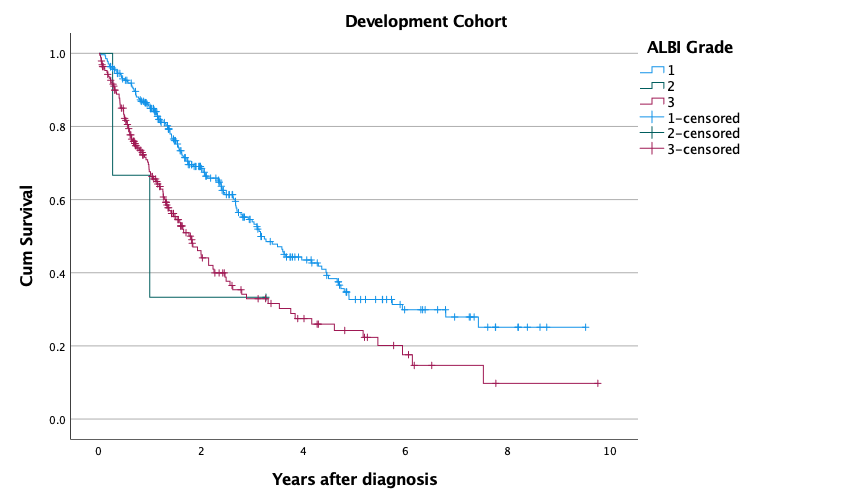


*P <.0001*


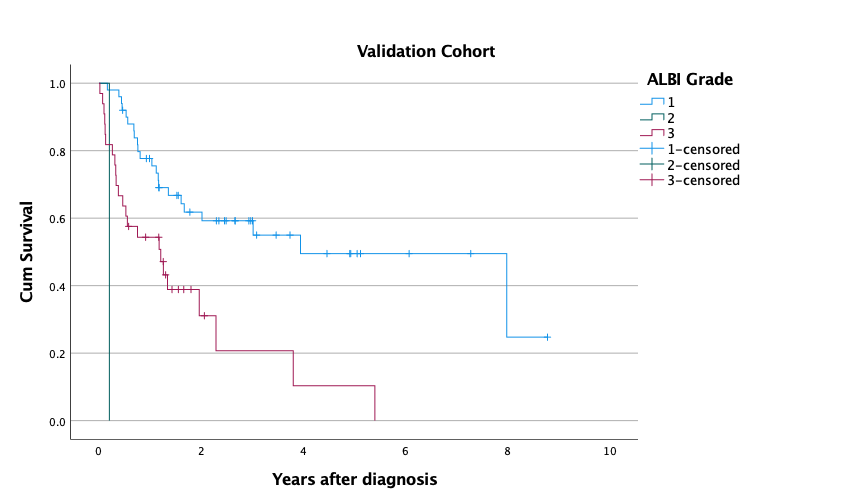


*P* <.0001

Figure S2.01. MRI co-registration example: A randomly selected subject from the validation cohort is presented with axial pre-contrast, arterial, portal-venous, and delayed phase contrast-enhanced magnetic resonance images. All images are co-registered to the subject’s portal-venous phase imaging. Automated liver segmentations in portal-venous phase imaging are overlaid in blue for reference in all phases to demonstrate accurate co-registration both within the liver and in the surrounding anatomy.

Figure S2.02. MRI co-registration example: A randomly selected subject from the validation cohort is presented with axial pre-contrast, arterial, portal-venous, and delayed phase contrast-enhanced magnetic resonance images. All images are co-registered to the subject’s portal-venous phase imaging. Automated liver segmentations in portal-venous phase imaging are overlaid in blue for reference in all phases to demonstrate accurate co-registration both within the liver and in the surrounding anatomy.

Figure S2.03. MRI co-registration example: A randomly selected subject from the validation cohort is presented with axial pre-contrast, arterial, portal-venous, and delayed phase contrast-enhanced magnetic resonance images. All images are co-registered to the subject’s portal-venous phase imaging. Automated liver segmentations in portal-venous phase imaging are overlaid in blue for reference in all phases to demonstrate accurate co-registration both within the liver and in the surrounding anatomy.

**Supplemental Material References**

1 Li X, Morgan PS, Ashburner J, Smith J, Rorden C (2016) The first step for neuroimaging data analysis: DICOM to NIfTI conversion. J Neurosci Methods 264:47-56

2 X. Papademetris MJ, N. Rajeevan, H. Okuda, R.T. Constable, L.H Staib BioImage Suite: An integrated medical image analysis suite, Section of Bioimaging Sciences, Dept. of Diagnostic Radiology, Yale School of Medicine. <http://www.bioimagesuite.org>.

3 Rueckert D, Sonoda LI, Hayes C, Hill DLG, Leach MO, Hawkes DJ (1999) Nonrigid Registration Using Free-Form Deformations: Application to Breast MR Images. IEEE Trans Med Imaging 18:712–721

4 Studholme C, Hill DL, Hawkes DJ (1999) An overlap invariant entropy measure of 3D medical image alignment. Pattern Recognition 32:71-86

5 Gross M, Huber S, Arora S et al (2024) Automated MRI liver segmentation for anatomical segmentation, liver volumetry, and the extraction of radiomics. Eur Radiol. 10.1007/s00330-023-10495-5

6 van Griethuysen JJM, Fedorov A, Parmar C et al (2017) Computational Radiomics System to Decode the Radiographic Phenotype. Cancer Res 77:e104-e107

7 Pyradiomics-community (February 11, 2020) pyradiomics Documentation Release v3.0. Available via <https://pyradiomics.readthedocs.io/en/v3.0/features.html>. Accessed 2023 February 24

8 Child CG, Turcotte JG (1964) Surgery and portal hypertension. Major Probl Clin Surg 1:1-85

9 Reig M, Forner A, Rimola J et al (2022) BCLC strategy for prognosis prediction and treatment recommendation: The 2022 update. J Hepatol 76:681-693

10 Yau T, Tang VY, Yao TJ, Fan ST, Lo CM, Poon RT (2014) Development of Hong Kong Liver Cancer staging system with treatment stratification for patients with hepatocellular carcinoma. Gastroenterology 146:1691-1700

11 Amin MB, Greene FL, Edge SB et al (2017) The Eighth Edition AJCC Cancer Staging Manual: Continuing to build a bridge from a population-based to a more "personalized" approach to cancer staging. CA Cancer J Clin 67:93-99

12 Japan TLCSGo (1989) The general rules for the clinical and pathological study of primary liver cancer. Jpn J Surg 19:98-129

13 Kudo M, Chung H, Osaki Y (2003) Prognostic staging system for hepatocellular carcinoma (CLIP score): its value and limitations, and a proposal for a new staging system, the Japan Integrated Staging Score (JIS score). J Gastroenterol 38:207-215

14 Johnson PJ, Berhane S, Kagebayashi C et al (2015) Assessment of liver function in patients with hepatocellular carcinoma: a new evidence-based approach-the ALBI grade. J Clin Oncol 33:550-558
